# Feasibility and Implementation of a Deep Learning MR Reconstruction for TSE Sequences in Musculoskeletal Imaging

**DOI:** 10.3390/diagnostics11081484

**Published:** 2021-08-16

**Authors:** Judith Herrmann, Gregor Koerzdoerfer, Dominik Nickel, Mahmoud Mostapha, Mariappan Nadar, Sebastian Gassenmaier, Thomas Kuestner, Ahmed E. Othman

**Affiliations:** 1 Department of Diagnostic and Interventional Radiology, Eberhard Karls University Tuebingen, Hoppe-Seyler-Strasse 3, 72076 Tuebingen, Germany; judith.herrmann@med.uni-tuebingen.de (J.H.); sebastian.gassenmaier@med.uni-tuebingen.de (S.G.); thomas.kuestner@med.uni-tuebingen.de (T.K.); 2 MR Applications Predevelopment, Siemens Healthcare GmbH, Allee am Roethelheimpark 2, 91052 Erlangen, Germany; gregor.koerzdoerfer@siemens-healthineers.com (G.K.); marcel.nickel@siemens-healthineers.com (D.N.); 3 Digital Technology & Innovation, Siemens Medical Solutions USA, Inc., Princeton, NJ 08540, USA; Mahmoud.Mostapha@siemens-healthineers.com (M.M.); mariappan.nadar@siemens-healthineers.com (M.N.); 4 Department of Neuroradiology, University Medical Center, 55131 Mainz, Germany

**Keywords:** magnetic resonance imaging, deep learning reconstruction, image processing, musculoskeletal imaging

## Abstract

Magnetic Resonance Imaging (MRI) of the musculoskeletal system is one of the most common examinations in clinical routine. The application of Deep Learning (DL) reconstruction for MRI is increasingly gaining attention due to its potential to improve the image quality and reduce the acquisition time simultaneously. However, the technology has not yet been implemented in clinical routine for turbo spin echo (TSE) sequences in musculoskeletal imaging. The aim of this study was therefore to assess the technical feasibility and evaluate the image quality. Sixty examinations of knee, hip, ankle, shoulder, hand, and lumbar spine in healthy volunteers at 3 T were included in this prospective, internal-review-board-approved study. Conventional (TSE_S_) and DL-based TSE sequences (TSE_DL_) were compared regarding image quality, anatomical structures, and diagnostic confidence. Overall image quality was rated to be excellent, with a significant improvement in edge sharpness and reduced noise compared to TSE_S_ (*p* < 0.001). No difference was found concerning the extent of artifacts, the delineation of anatomical structures, and the diagnostic confidence comparing TSE_S_ and TSE_DL_ (*p* > 0.05). Therefore, DL image reconstruction for TSE sequences in MSK imaging is feasible, enabling a remarkable time saving (up to 75%), whilst maintaining excellent image quality and diagnostic confidence.

## 1. Introduction

Magnetic Resonance Imaging (MRI) has become an important diagnostic tool for the evaluation of the musculoskeletal (MSK) system. Due to its high clinical impact, MSK imaging is one of the most common performed examinations in clinical routine. One big disadvantage of MRI is the long examination time, which is not tolerated by a substantial proportion of patients and, on the other hand, comes along with other downsides such as decreased image quality due to motion artifacts, increased costs and reduced patient throughput [1].

The acquisition time of MRI is primarily determined by the achievable sampling rate for a given contrast and image quality, which, in turn, is determined by the number of samples needed for the image reconstruction for a given size and resolution. Over the past decades, different acceleration strategies have been proposed and established such as parallel imaging (PI) and Compressed Sensing (CS) [2,3,4,5,6,7,8,9,10,11,12,13,14]. These techniques acquire a reduced amount of k-space data with an array of receiver coils and afterwards reconstruct images from the acquired, under sampled data [2,14]. Artifacts due to residual aliasing, noise, or stair casing and blurring can impair achievable image quality of those acceleration strategies.

Recently, a new acceleration strategy gained attention: Deep Learning (DL) reconstruction, as discussed here and detailed below, may solve nonlinear and ill-posed reconstruction problems efficiently [15,16,17]. Instead of ad hoc regularization that enforces sparsity, the regularization is trained on representative images. This procedure also allows the regularization to generalize for different sampling patterns, acceleration factors, and artifact behavior. With DL reconstruction providing higher signal-to-noise ratio (SNR) and allowing for higher acceleration with conventional sampling patterns, the data acquisition can be tailored. For TSE acquisitions, modifying the original acquisition’s image contrast is usually not a desirable goal for tailored acquisitions. Rather, DL reconstruction in MRI can be used to improve on a combination of image resolution, acquisition time, and SNR while maintaining the original contrast [18,19].

Most prominently, the DL networks are trained in a supervised manner, i.e., representative fully sampled data with known results for the given application—so-called ground truth data—are available, which allow retrospective subsampling and training of the architectures. Testing is performed on a separate set of samples (not seen in training) with subsampled datasets. While training can be computationally intensive and takes rather long, it can be performed offline. The trained architecture can then be used in testing to reconstruct an aliasing and noise-free image within a few seconds and with greatly reduced computational demand [20,21,22].

DL reconstruction has recently been shown to potentially accelerate image acquisition in knee MRI [23]; however, the majority of existing literature on this topic focusses on offline solutions simulating accelerated image acquisition. Realistic performance assessment should be performed on prospectively subsampled datasets. To evaluate the clinical impact of the DL reconstruction for MSK imaging, an implementation in clinical settings using a prospective acquisition of accelerated data, as well as an extension to various joints, is needed.

Therefore, the aim of this study was to first assess the technical feasibility of the DL reconstruction, and to compare the obtainable image quality for accelerated DL-based TSE sequences (TSE_DL_) in comparison to conventional TSE sequences (TSE_S_) for the most common MSK examinations in healthy volunteers. In addition, technical guidance is provided for prospective implementation of DL image reconstruction into clinical workflow.

## 2. Materials and Methods

### 2.1. Study Design

Institutional review board approval was obtained for this prospective, monocentric study (Eberhard Karls University Tuebingen, project identification code: 055/2017BO2, 2 December 2020). All volunteers gave informed consent to participate in this study and all study procedures were conducted in accordance with the ethical standards as laid down in the 1964 Declaration of Helsinki and its later amendments.

A total of 60 volunteer-MRI-examinations, ten from each joint (ankle, hand, hip, knee, lumbar spine, and shoulder) were included in this study.

A short protocol comprising PD-weighted TSE sequences with fat suppression (FS) of the two clinically most commonly used orientations for ankle (axial/sagittal), hand (axial/coronal), hip (axial/coronal), knee (coronal/sagittal) and shoulder (axial/coronal), as well as T2-weighted and T1-weighted TSE sequences with FS for the lumbar spine in sagittal orientation were acquired. Accelerated TSE_DL_ sequences were acquired along with standard TSE_S_ sequences at clinical 3 T MRI scanner (MAGNETOM Prisma^fit^, Siemens Healthcare, Erlangen, Germany) using clinical surface coils respectively for each joint. An overview of the acquisition parameters is displayed in Table 1.

### 2.2. Deep Learning Image Reconstruction

**Figure 1:**
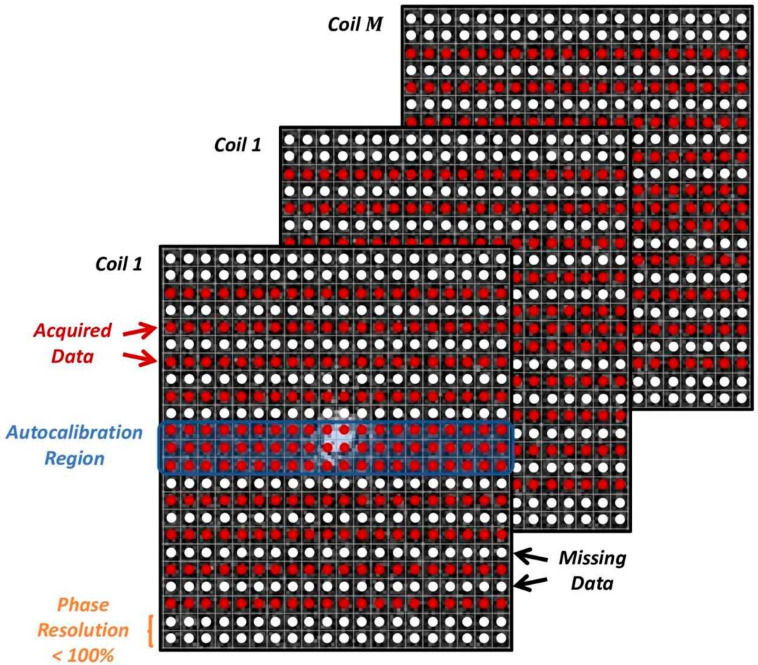
Exemplary sampling pattern used for the k-space data acquisition (red arrows point to the acquired date; black arrows point to the missing data). Along the phase encoding direction, data are under sampled by an acceleration factor R. An autocalibration region covering the center of k-space is acquired, either along with the imaging acquisition or separately with a different image contrast. A fraction of the periphery may be skipped, usually referred to as phase resolution.

A conventional under sampling pattern, as known from parallel imaging, is used. As shown in earlier works [24,25], these provide the same performance when reconstructed with DL-based methods as incoherent sampling patterns favored by CS. They have the important advantages of being clinically established and are highly flexible regarding adaptations of resolution and signal evolution during sampling. Furthermore, their artifact behavior with regard to motion, reduced field of view, and aliasing is well known and potentially even improved by the DL-based reconstruction. In addition, as a part of conventional under sampling patterns, a fraction of the k-space’s periphery is often not acquired in order to reduce acquisition time. This effectively reduces the resolution in the phase-encoding direction and is referred to as phase resolution. It describes the fraction of acquired data in the phase-encoding direction in percent neglecting the regular parallel imaging type of under sampling. For illustration, an exemplary sampling pattern of acceleration factor 2 and a phase resolution <100% is shown in Figure 1.

Besides the data acquisition for the actual image data, calibration data for the coil-sensitivity estimation needs to be acquired. For the TSE sequence, a fully sampled region around the k-space center (typically about 16 phase-encoding lines) is acquired as part of the imaging scan. These data are used for both the image reconstruction and the estimation of coil sensitivity maps.

**Figure 2:**
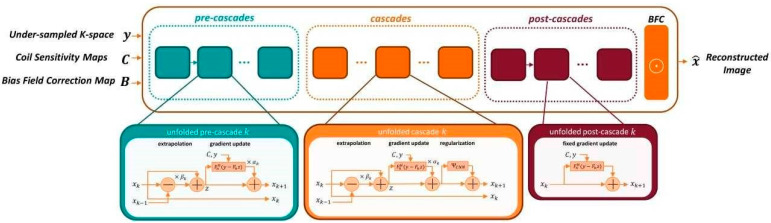
Architecture of the fixed iterative reconstruction scheme. Pre-cascades address data consistency and generate an image similar to conventional parallel imaging, cascades use an alternating scheme between data consistency and regularization, post-cascades reemphasize consistency of a reconstructed image and acquired data. Note that only the regularization is a Convolutional Neural Network (CNN). Nevertheless, the whole architecture can be presented by a general network.

For all discussed applications, the prototype image reconstruction comprises a fixed iterative reconstruction scheme or variational network [25,26]. The deep neural network model leverages the combined benefits of physical models of MR imaging (SENSE model with coil sensitivity maps [12] with bias field corrections) and data-driven models. The fixed unrolled algorithm for accelerated MR image reconstruction consists of multiple cascades (6 pre-cascades, 12 cascades, and 1 post-cascade), each made up from a data consistency using a trainable Nesterov Momentum followed by a CNN-based regularization. The regularization model’s architecture is based on a novel hierarchical design of an iterative network that repeatedly decreases and increases the resolution of the feature maps, allowing for a more memory-efficient model than conventional CNNs. The CNN module is a “Deep, Iterative, Hierarchical Network” that extends the Down-Up network [27] with a hierarchical block design, facilitating memory efficiency over a standard U-Net [28]. In addition to the input under sampled k-space data, coil-sensitivity maps are also provided, which are estimated from the calibration data as a non-DL based pre-processing step. In addition, a bias field is extracted from a separate adjustment acquisition for image homogenization. The architecture of the reconstruction network is illustrated in Figure 2. During the training phase, the bias field is inserted into the image reconstruction as a final correction step.

For the image reconstruction, the k-space data, the bias field and coil-sensitivity maps are inserted into the variational network. Compared to previous works and the previously described cascades, the variational network also utilizes two additional types of cascades, namely, pre- and post-cascades. Like regular cascades, pre-cascades employ trainable extrapolation; however, no regularization is applied, allowing the network to focus on the parallel imaging part of the reconstruction problem. Such design is motivated by the empirical finding that initial steps in the variational network focus on the signal recovery of missing data near the k-space center. This approach supports acquisitions without integrated calibration and flexible k-space sampling. Finally, post-cascades employing non-trainable extrapolation are also utilized for further guarantees on the data consistency, which minimizes the risk of hallucination when adversarial training is applied. The network is first trained to minimize the combined L1 and a multi-scale version of the structural similarity (SSIM) content losses between network prediction and ground truth images [29].

The reconstruction was trained on volunteer acquisitions using conventional TSE protocols independently of the data acquired in this study. About 10,000 slices were acquired on volunteers using clinical 1.5 T and 3 T scanners (MAGNETOM scanners, Siemens Healthcare, Erlangen, Germany). Fully sampled acquisitions with high resolution were performed in head, pelvis, and knee using representative contrasts for the respective body regions. The training data therefore included a wide range of image contrasts, orientations, body regions, and resolutions. The input to the reconstruction network was retrospectively under sampled to an acceleration factor of 4. The training was implemented in PyTorch and performed on a GPU cluster NVIDIA Tesla V100 (32 GB of memory) GPU.

### 2.3. Implementation of DL Image Reconstruction in Clinical Workflow

For deployment in the scanner reconstruction pipeline, the obtained network was converted to a C++ implemented inference framework. For the CPU-only reconstruction on a clinical MRI scanner, inference needed about 4 s per slice for the used protocol settings. The reconstruction was triggered after the end of the acquisition, which resulted in a perceived reconstruction time of 2–3 min including additional pre- and post-processing. GPU-based reconstruction can bring the duration down to the order of 20 s for a complete dataset but was not available in the local setting of this study.

### 2.4. Image Analysis

Image analysis was performed by two radiologists with three and nine years of experience. Both readers independently analyzed both TSE_S_ and TSE_DL_ datasets by using a random order. The radiologists assessed qualitative image parameters including overall image quality, diagnostic confidence, edge sharpness, contrast resolution, as well as the extent of noise and artifacts using an ordinal 4-point Likert scale (1 = non-diagnostic, with major streak artifacts; 2 = non-diagnostic, moderate artifacts with low image quality; 3 = minor artifacts with good image quality; 4 = no artifacts with excellent image quality). Complementary, the overall clarity of anatomical structures, the assessment of bone and articular cartilage, as well as the delineation of ligaments and tendons were assessed as well on a 4-point Likert scale (4 = excellent clarity of the anatomical structures/assessment of bone and articular cartilage; 3 = good; 2 = acceptable; 1 = poor). Furthermore, the subjective image impression was evaluated by both readers (1 = very unrealistic, 2 = unrealistic, 3 = realistic, and 4 = very realistic). Reading scores were considered sufficient when reaching ≥3.

Image analysis was performed on a PACS workstation (GE Healthcare Centricity™ PACS RA1000, Milwaukee, WI, USA).

### 2.5. Statistical Analysis

Statistical analyses were performed using SPSS version 26 (IBM Corp, Armonk, NY, USA). Besides descriptive statistics, compromising median and interquartile range (IQR), reading scores of the qualitative image analysis of the TSE sequences were compared using a Wilcoxon signed-rank test for paired data of ordinal structure. Significance was assumed at a level of *p* < 0.05.

Inter-rater agreement was calculated through Cohen’s kappa. Kappa values were interpreted as follows: 0–0.20 = poor agreement, 0.21–0.40 = fair agreement, 0.41–0.60 = moderate agreement, 0.61–0.80 = substantial agreement, 0.81–1 = (almost) perfect agreement.

## 3. Results

The aim of this work was to assess the technical feasibility of the DL image reconstruction for TSE sequences in MSK imaging. In total, 60 examinations in healthy volunteers (37 males, age: 26 ± 7 (20–55) years) were included in this prospective study, ten from each MSK region (knee, ankle, hip, shoulder, hand, lumbar spine), as shown in Table 2.

All TSE_DL_ were successfully acquired for all regions. Fat suppression could be applied successfully for TSE_DL_. TSE_DL_ allowed a remarkable time saving by at least 21% and up to 75%; for instance, T1-weighted TSE in sagittal orientation for lumbar spine required an acquisition time of 2:45 min compared to T1-weighted TSE_DL_ with an acquisition time of 0:58 min, or TSE_S_ of the hand in coronal orientation required 2:23 min compared to 0:36 min for TSE_DL_ (see Table 1).

As inter-reader agreement resulted in a Cohen’s kappa of 0.634 to 0.923, the results of the more experienced reader 1 are described in the following.

### 3.1. Assessment of Image Quality

**Figure 3:**
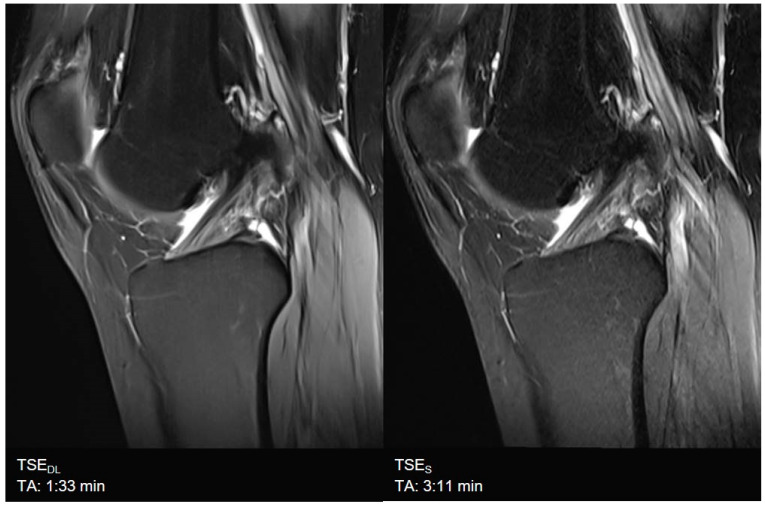
Example of a Deep Learning and standard PD-weighted turbo spin echo (TSE) image of the knee in sagittal orientation. Note that the extent of noise in TSE_DL_ (**left**) is distinctly reduced compared to TSE_S_ (**right**), which comes with the fact that, at the same time, very small structures such as small bone channels disappear. The visibility of anatomic relevant structures is not impaired.

**Figure 4:**
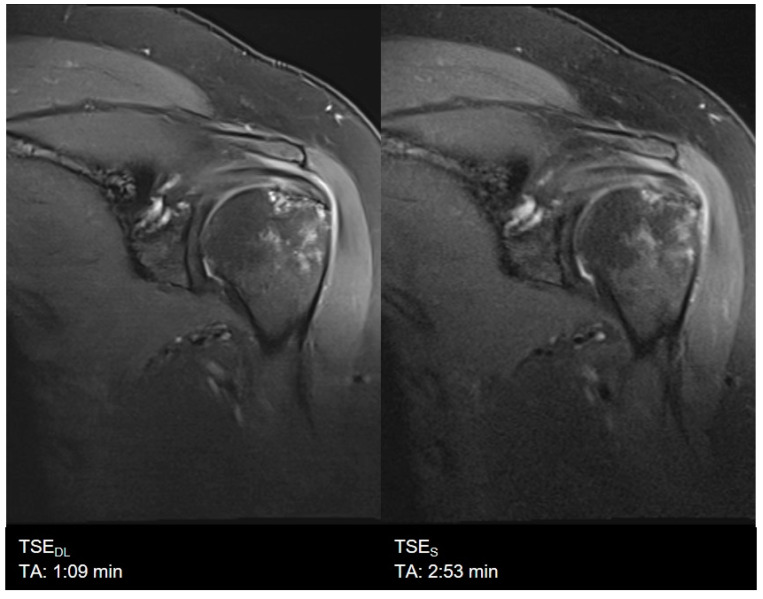
Example of a Deep Learning (**left**) and standard (**right**) PD-weighted turbo spin echo image of the shoulder in coronal orientation.

**Figure 5:**
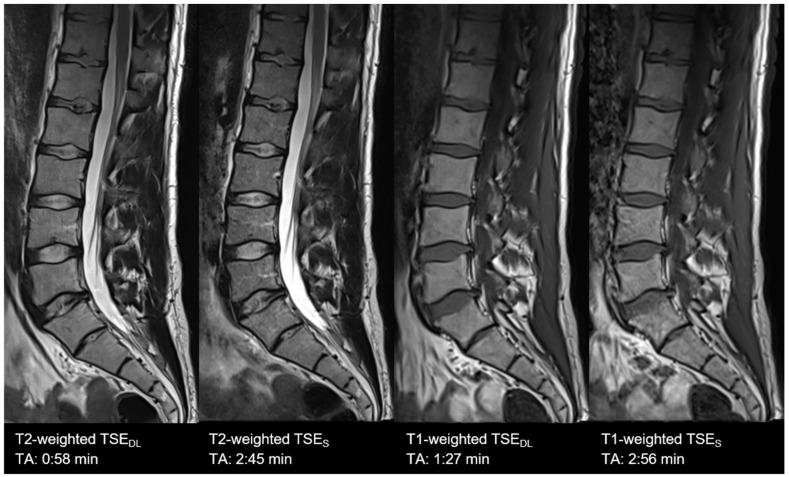
Example of a Deep Learning and standard T1- (**right**) and T2-weighted (**left**) turbo spin echo image of the lumbar spine in sagittal orientation. Note that TSE_DL_ show lower extents of noise both in T1- and T2-weighted imaging. Nonetheless, some small structures, such as small bone canals, disappear; there is no impact on the delineation and assessment of relevant anatomical structures in both TSE_S_ and TSE_DL_.

**Figure 6:**
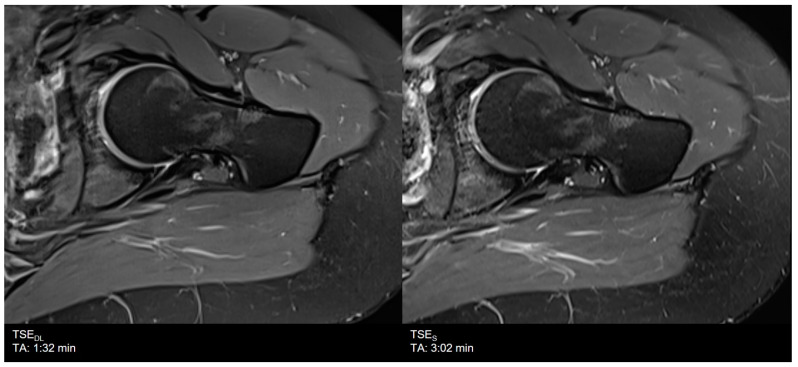
Example of a Deep Learning (**left**) and standard (**right**) PD-weighted turbo spin echo image of the hip in axial orientation. Note that although the assessment of the bone was rated to be lower, the assessment of anatomical structures and articular cartilage, as well as the delineation of ligaments and tendons, are comparable between TSE_DL_ and TSE_S_.

**Figure 7:**
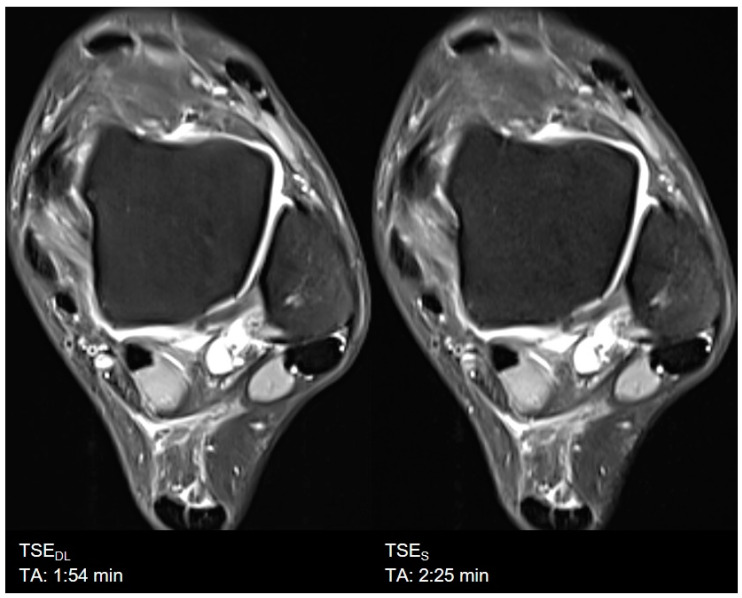
Example of a Deep Learning (**left**) and standard (**right**) PD-weighted turbo spin echo image of the ankle in axial orientation.

**Figure 8:**
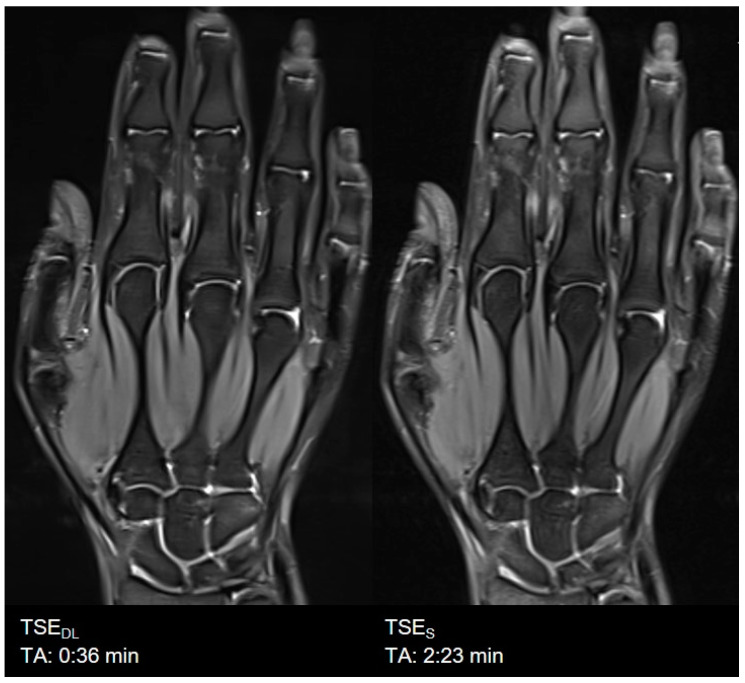
Example of a Deep Learning (**left**) and standard (**right**) PD-weighted turbo spin echo image of the hand in coronal orientation.

**Figure 9:**
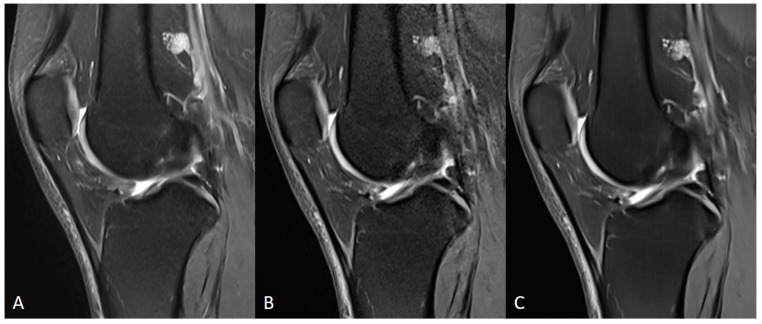
Comparison of TSE_S_ (**A**), TSE_DL_ raw data reconstructed with a standard (GRAPPA) method (**B**) and TSE_DL_ (**C**) of a knee in sagittal orientation. Note that the extent of noise in TSE_DL_ with standard reconstruction (**B**) is increased compared to TSE_S_ (**A**) and TSE_DL_.

**Figure 10:**
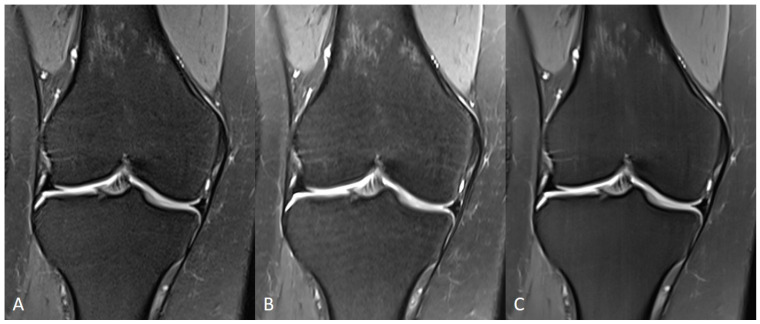
Comparison of TSE_S_ (**A**), TSE_DL_ raw data reconstructed with a standard (GRAPPA) method (**B**) and TSE_DL_ (**C**) of a knee in coronal orientation. Note that the image quality in TSE_DL_ (**C**) is increased compared to TSE_S_ (**A**) and TSE_DL_ with standard reconstruction (**B**).

**Figure 11:**
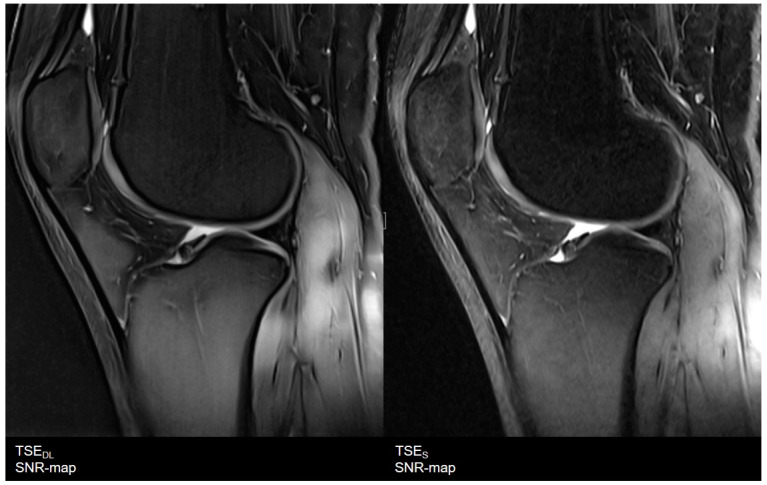
Exemplary visualization of signal-to-noise ratio (SNR) as SNR-maps. On the (**left**), results of the TSE_DL_ dataset, and on the (**right**), of a TSE_S_ dataset of knee in sagittal orientation. The TSE_DL_ dataset shows lower noise levels and an increase of SNR with a more homogeneous distribution compared to the TSE_S_ dataset. In addition, SNR levels throughout the whole image are more homogeneous.

Overall image quality was rated to be excellent for TSE_DL_ (median 4, IQR 4−4), significantly higher compared to TSE_S_ (median 4, IQR 4−4, *p* = 0.002) with significantly lower levels of noise (TSE_S_: median 4, IQR 3−4; TSE_DL_: median 4, IQR 4−4; *p* < 0.001) and improved edge sharpness (TSE_S_: median 4, IQR 3−4; TSE_DL_: median 4, IQR 4−4; *p* = 0.008) in TSE_DL_. No difference was found for contrast resolution and the extent of artifacts, which were both rated as excellent in TSE_S_ (median 4, IQR 4−4) and TSE_DL_ (median 4, IQR 4−4, *p* > 0.05). Image examples are shown in Figure 3, Figure 4, Figure 5, Figure 6, Figure 7 and Figure 8. To highlight the image quality difference of the DL-reconstruction compared to standard reconstruction, we exported raw data of TSE_DL_ of a single volunteer and reconstructed the accelerated TSE_DL_ raw data with the standard reconstruction method (GRAPPA) as used in TSE_S_ (see Figure 9 and Figure 10). To illustrate the effect of the DL reconstruction on the extent of noise, raw data of a single volunteer were exported, and exemplary SNR-maps were determined offline using a pseudo-replica method [30]; see Figure 11.

### 3.2. Assessment of Anatomical Structures

The overall clarity of anatomic structures was rated excellent for TSE_DL_ (median 4, IQR 4−4) and comparable to TSE_S_ (*p* > 0.05). Furthermore, the assessment of the articular cartilage, the delineation of ligaments and tendons were all rated excellent and to be comparable between the two sequences (median 4, IQR 4−4, *p* > 0.05). The assessment of the bone was rated to be lower in TSE_DL_ (median 4, IQR 4−4) compared to TSE_S_ (median 4, IQR 4−4). There was no significant difference regarding the diagnostic confidence between both sequences (median 4, IQR 4−4, *p* > 0.05).

Subjective image impression was rated to be more realistic in TSE_S_ (median 4, IQR 4−4) compared to TSE_DL_ (median 4, IQR 3−4, *p* < 0.001). An overview of all results is displayed in Table 3.

## 4. Discussion

This study investigated the technical feasibility and impact of a DL image reconstruction for TSE sequences in MSK MRI in a prospective volunteer study compared to standard TSE imaging. The results demonstrate that TSE with DL image reconstruction is feasible, providing even higher image quality as well as an improvement in edge sharpness, and concerning the evaluation of MSK-specific items, an equivalent assessment of the articular cartilage, correspondingly equal delineation of ligaments and tendons. Therefore, TSE with DL image reconstruction maintains equal diagnostic confidence compared to conventional TSE.

The fact that DL-based reconstructed images exhibit even lower noise levels than corresponding fully sampled conventional acquisition techniques, and may therefore look artificial to experienced radiologists, was already discussed in prior studies [15,23] and is in line with our findings: TSE_S_ is rated to have a more realistic image impression compared to TSE_DL_. The low extent of noise may also be the cause of the lower ratings of the assessment of the bone, due to almost noise-free image impression, in which very small structures disappear. Nonetheless, this is only an impairment of the image impression and there is no impact on the diagnostic accuracy since TSE_DL_ exhibited excellent diagnostic confidence.

Another aim of the study was to accelerate TSE sequence acquisition by the incorporation of DL image reconstruction. DL allowed for an acquisition time reduction of up to 50% while maintaining excellent image quality and diagnostic confidence. DL therefore seems to allow for higher accelerations than prior acceleration techniques. Prior to DL image reconstruction, high acceleration levels beyond the Nyquist–Shannon sampling limit could be obtained by CS. In fact, if images can be sparsely represented in some transform domain, then a random and incoherent sub-Nyquist sampling together with an appropriate nonlinear iterative image reconstruction allows aliasing-free recovery from incompletely sampled k-space data [14]. CS employs iterative reconstruction algorithms that use a priori fixed sparsity-promoting transformation. Furthermore, the a priori assumption on sparsity with application-specific regularization weighting can, if not chosen appropriately, result in residual aliasing (under-regularized) or stair casing and blurring (over-regularized). Depending on the imaging application and sequence, a sampling trajectory that follows a desired random distribution can be challenging to implement without introducing other artifacts, e.g., by eddy currents due to strong switching gradients. Overall, these factors can impair achievable image quality and/or limit the achievable acceleration. The implementation of DL can overcome this drawback and enables the acceleration of MR acquisition without impairing image quality.

Current Deep Learning-based image reconstruction uses supervised learning techniques with convolutional neural networks (CNNs) [24,31]. DL networks have been proposed that operate on parallel imaging (PI)-accelerated acquisitions [25,26,32] and on CS-accelerated acquisitions [33]. The proposed methods primarily differ in the way the DL network is applied in the image reconstruction and how data consistency is enforced between reconstructed images and acquired data: (i) the network acts as trainable denoiser without explicit data consistency inside the architecture during training, but handling it in outer optimization schemes (plug-and-play denoisers), (ii) physics-based reconstructions that incorporate data consistency during training. Networks furthermore differ in the chosen architectures (VN, UNet, cascaded CNN, etc.), raw k-space or noisy/aliased image input, the input dimensionality (2D, 3D, 2D+time, 3D+time, etc.), single- or multi-parametric input, complex- or real-valued processing of the complex-valued data, and single-coil (coil-combined) or multi-coil processing [20,21,25,26,33,34,35,36,37,38,39,40,41,42,43]. To date, there are only a small number of published studies using DL methods on k-space data for MR image reconstruction in a clinical setting. Although the results are promising, routine application in clinical practice remains rare. As we successfully implemented DL reconstruction in imaging of different body regions, this report is intended to motivate radiologists to establish new AI techniques in everyday clinical practice to further accelerate MR imaging and gain access to MRI for more patients. Prolonged scanning duration can cause patient inconvenience and also limits the availability of MRI, which makes the single examination slots expansive. Drastic acceleration and cut-down of examination times might display one piece of the big puzzle to enhance health care and balance the weight of supply and demand. One of the most challenging tasks in medicine has always been finding a compromise between best medical care and best economical outcome. As most healthcare systems worldwide are insurance-based using different architectures (mandatory versus voluntary; public health insurance versus private sector), all systems face the same problem: shortage of money versus increased demand due to the development of new expensive therapies, increase in expectancy of life, and new diagnostic possibilities. Reduction of TA might allow for a higher number of examinations per day.

This study has limitations. First, we included a small amount of image data acquired in healthy volunteers. Second, we just investigated the TSE_DL_ at 3 T; further studies are needed to evaluate its feasibility and diagnostic confidence at 1.5 T. This impacts the generalizability of our findings. However, this report primarily aims to describe the implementation of DL-based reconstruction and to assess its technical feasibility rather than to systematically and comprehensively evaluate the resulting image quality in routine clinical practice. For image quality assessment, body-region-focused clinical studies at 1.5 and 3 T are still required. A further limitation is the fact that DL-based reconstruction was not applied on 3D MR sequences yet. DL-based reconstruction algorithms for 3D sequences are still being developed by our team.

To conclude, DL image reconstruction can be implemented in clinical workflow and enables accelerated image acquisition, allowing a remarkable time saving while maintaining excellent image quality for TSE sequences.

## Figures and Tables

**Table 1:** Acquisition parameters of TSE_S_ and TSE_DL_ at 3 T.

		Sequence	Orientation	TA, min	FOV, mm	Voxel Size, mm	A	C	PAT	TR, ms	TE, ms	FA, Degree	Bandwith, Hz/Px	Echo Spacing, ms
Shoulder	TSE_S_	TSE PD FS	axial	2:14	180	0.6 × 0.6 × 3.0	1	2	2	3000	44	150	180	10.9
			coronal	2:53	180	0.6 × 0.6 × 3.0	2	1	2	3300	42	150	191	10.6
	TSE_DL_	TSE PD FS	axial	1:10	180	0.6 × 0.6 × 3.0	1	1	3	3520	44	150	180	10.9
			coronal	1:09	180	0.6 × 0.6 × 3.0	1	1	3	3000	42	150	191	10.6
Knee	TSE_S_	TSE PD FS	coronal	3:11	150	0.2 × 0.2 × 3.0	2	1	3	3790	44	150	100	14.6
			sagittal	3:11	150	0.2 × 0.2 × 3.0	2	1	3	3790	44	150	100	14.6
	TSE_DL_	TSE PD FS	coronal	1:33	150	0.5 × 0.5 × 3.0	1	1	3	3580	41	150	120	13.7
			sagittal	1:33	150	0.5 × 0.5 × 3.0	1	1	3	3580	41	150	120	13.7
Lumbar spine	TSE_S_	T1 TSE	sagittal	2:56	300	0.9 × 0.9 × 3.0	1	2	0	562	10	150	180	10.4
		T2 TSE FS	sagittal	2:45	300	0.7 × 0.7 × 3.0	2	1	2	6040	102	150	189	11.3
	TSE_DL_	T1 TSE	sagittal	1:27	300	0.9 × 0.9 × 3.0	1	2	3	462	10	150	180	10.4
		T2 TSE FS	sagittal	0:58	300	0.7 × 0.7 × 3.0	1	1	3	4470	105	150	189	10.5
Hip	TSE_S_	TSE PD FS	axial	3:02	200	0.3 × 0.3 × 3.0	1	1	0	3410	42	150	100	14.1
			coronal	2:01	200	0.3 × 0.3 × 3.0	1	1	2	3410	42	150	100	14.1
	TSE_DL_	TSE PD FS	axial	1:32	200	0.6 × 0.6 × 3.0	1	1	3	3069	42	150	120	13.1
			coronal	1:33	200	0.6 × 0.6 × 3.0	1	1	3	3000	41	150	120	13.7
Ankle	TSE_S_	TSE PD FS	axial	2:25	150	0.4 × 0.4 × 3.0	1	1	2	3340	17	150	90	17.1
			sagittal	1:47	160	0.2 × 0.2 × 3.0	1	1	3	3000	32	150	100	15.9
	TSE_DL_	TSE PD FS	axial	1:54	150	0.4 × 0.4 × 3.0	1	1	3	3000	17	150	90	16.9
			sagittal	1:45	160	0.4 × 0.4 × 3.0	1	1	3	3000	31	150	100	15.7
Hand	TSE_S_	TSE PD FS	coronal	2:23	200	0.5 × 0.5 × 2.0	2	1	0	3000	41	150	121	13.6
			axial	4:40	180	0.5 × 0.5 × 2.0	2	2	0	3310	42	150	121	13.9
	TSE_DL_	TSE PD FS	coronal	0:36	200	0.5 × 0.5 × 2.0	1	1	3	3000	44	150	119	14.7
			axial	1:23	180	0.5 × 0.5 × 2.0	1	2	2	3190	42	150	119	14.1

TA indicates time of acquisition; FOV, field of view; A, Averages; C, Concatenations; PAT, Parallel Acquisition Technique; TE/TR, echo time/repetition time; FA, flip angle; TSE, turbo spin echo; PD, Proton Density; FS, fat saturation.

**Table 2:** Demographics.

Variables	
Total (male/female), *n*	60 (37/23)
Age, mean ± SD (range), y	total: 26 ± 7 (20–55)
	knee: 25 ± 4 (20–31)
	ankle: 26 ± 5 (20–35)
	hip: 26 ± 5 (20–35)
	shoulder: 27 ± 10 (20–55)
	wrist: 27 ± 8 (20–44)
	lumbar spine: 29 ± 11 (20–55)

SD indicates standard deviation; y, years; *n*, number.

**Table 3:** Image Quality, Inter-reader Agreement and Comparison of TSE and TSE_DL_.

	TSE_S_			TSE_DL_			TSE_S_ vs. TSE_DL_	
	R1 m (IQR)	R2 m (IQR)	κ	R1 m (IQR)	R2 m (IQR)	κ	R1	R2
IQ	4 (4−4)	4 (3−4)	0.697	4 (4−4)	4 (4−4)	0.634	0.002	0.013
Artifacts	4 (4−4)	4 (4−4)	0.649	4 (4−4)	4 (4−4)	0.700	0.180	0.157
Edge sharpness	4 (3−4)	4 (3−4)	0.883	4 (4−4)	4 (4−4)	0.792	<0.001	<0.001
Contrast resolution	4 (4−4)	4 (4−4)	0.741	4 (4−4)	4 (4−4)	0.649	0.257	0.157
Noise	4 (3−4)	4 (3−4)	0.897	4 (4−4)	4 (4−4)	0.651	<0.001	<0.001
Clarity of anatomic structures	4 (4−4)	4 (4−4)	0.747	4 (4−4)	4 (4−4)	0.889	0.317	0.564
Bone	4 (4−4)	4 (4−4)	0.896	4 (4−4)	4 (4−4)	0.741	0.014	0.025
Articular cartilage	4 (4−4)	4 (4−4)	0.739	4 (4−4)	4 (4−4)	0.773	0.157	0.705
Ligaments	4 (4−4)	4 (4−4)	0.732	4 (4−4)	4 (4−4)	0.643	0.705	0.480
Tendons	4 (4−4)	4 (4−4)	0.640	4 (4−4)	4 (4−4)	0.659	0.180	0.083
Diagnostic confidence	4 (4−4)	4 (4−4)	0.773	4 (4−4)	4 (4−4)	0.651	0.102	0.096
Image impression	4 (4−4)	4 (4−4)	0.848	4 (3−4)	4 (3−4)	0.923	<0.001	<0.001

IQ indicates Image Quality; R, Reader; m, median; κ, Cohen’s κ (Inter-rater Reliability); IQR, Interquartile Range.

## Data Availability

The datasets generated and analyzed during the current study are not publicly available to ensure data privacy protection but are available from the corresponding author on reasonable request.
